# A Preclinical Assessment of Neural Stem Cells as Delivery Vehicles for Anti-Amyloid Therapeutics

**DOI:** 10.1371/journal.pone.0034097

**Published:** 2012-04-04

**Authors:** eMalick G. Njie, Svetlana Kantorovich, Garrett W. Astary, Cameron Green, Tong Zheng, Susan L. Semple-Rowland, Dennis A. Steindler, Malisa Sarntinoranont, Wolfgang J. Streit, David R. Borchelt

**Affiliations:** 1 Department of Neuroscience, McKnight Brain Institute, University of Florida, Gainesville, Florida, United States of America; 2 Department of Biomedical Engineering, University of Florida, Gainesville, Florida, United States of America; 3 Department of Neurosurgery, University of Florida, Gainesville, Florida, United States of America; 4 Department of Mechanical and Aerospace Engineering, University of Florida, Gainesville, Florida, United States of America; 5 SantaFe HealthCare Alzheimer's Disease Research Center, University of Florida, Gainesville, Florida, United States of America; 6 Center for Translation Research in Neurodegenerative Disease, University of Florida, Gainesville, Florida, United States of America; Boston University School of Medicine, United States of America

## Abstract

Transplantation of neural stems cells (NSCs) could be a useful means to deliver biologic therapeutics for late-stage Alzheimer's disease (AD). In this study, we conducted a small preclinical investigation of whether NSCs could be modified to express metalloproteinase 9 (MMP9), a secreted protease reported to degrade aggregated Aβ peptides that are the major constituents of the senile plaques. Our findings illuminated three issues with using NSCs as delivery vehicles for this particular application. First, transplanted NSCs generally failed to migrate to amyloid plaques, instead tending to colonize white matter tracts. Second, the final destination of these cells was highly influenced by how they were delivered. We found that our injection methods led to cells largely distributing to white matter tracts, which are anisotropic conduits for fluids that facilitate rapid distribution within the CNS. Third, with regard to MMP9 as a therapeutic to remove senile plaques, we observed high concentrations of endogenous metalloproteinases around amyloid plaques in the mouse models used for these preclinical tests with no evidence that the NSC-delivered enzymes elevated these activities or had any impact. Interestingly, MMP9-expressing NSCs formed substantially larger grafts. Overall, we observed long-term survival of NSCs in the brains of mice with high amyloid burden. Therefore, we conclude that such cells may have potential in therapeutic applications in AD but improved targeting of these cells to disease-specific lesions may be required to enhance efficacy.

## Introduction

The extracellular deposition of the amyloid beta peptide (Aβ) is a requisite pathology for diagnosis of Alzheimer's disease (AD) [1], [2]. Aβ peptides also form dimers and oligomers that may play a role in inducing the neurodegeneration underlying AD-associated emotional, learning, and memory deficits [3]. Currently there are no effective therapies for AD. For the past two decades, most of the therapeutic focus on AD has been aimed at inhibiting or eliminating amyloid pathology. The focus on amyloid derives from genetic studies that have largely implicated amyloid deposition as a possible trigger in initiating disease [4]. Recent failures of clinical trials involving inhibitors of γ-secretase, which is critical in the generation of Aβ40 and 42 [5], have cast doubt on the potential efficacy of therapies targeting amyloid. Moreover, studies in mice expressing mutant amyloid precursor protein (APP), via promoters regulated by doxycycline, have demonstrated that cored Aβ plaques are highly stable structures that do not spontaneously resolve [6]. Recently, Wang et al demonstrated that amyloid plaque burden in this model can be partially ameliorated by passive transfer of antibody to Aβ peptide [7] suggesting that additional interventions may promote clearance of amyloid pathology. Current opinions on treatment of AD lean toward early therapeutic interventions, including delivery of inhibitors of γ- and, or, β-secretase [4]. While these approaches hold promise if issues of toxicity can be overcome, they offer little hope for patients with fulminant, late-stage disease.

The discovery of NSCs in the adult [8] and the development of *ex vivo* protocols to culture and re-introduce these cells into the central nervous system (CNS) [9], [10] have led to significant interest in using NSCs as possible vehicles for delivery of therapies for AD. In theory, it should be possible to obtain NSCs from the brains of patients with neurodegeneration, differentiate these NSCs to cell types of interest *in vitro*, manipulate them to express therapeutic biologics, and then re-introduce them to the patient's brain. The feasibility of this type of approach has recently been demonstrated by transplanting fibroblasts programmed to release nerve growth factor (NGF) [11] and neprilysin, a protease that degrades Aβ peptides, into the brain [12]. Most recently, Blurton-Jones and colleagues reported that NSCs transplanted into the hippocampus of transgenic mice that express mutant amyloid precursor protein, mutant presenilin 1, and mutant tau showed improved learning and memory by stimulating release of brain derived neurotrophic factor (BDNF) [13]. NSCs have also been reported to reduce neural inflammatory responses in rats whose brains had been injected with fibrillar forms of Aβ42 [14]. Together, the results of these studies support development of NSC-based therapies.

One of the major hurdles in developing NSC-based therapies is delivery of the cells to the site or sites of injury. In instances of acute brain pathology, endogenous and transplanted NSCs are reported to show migratory potential [15]–[18]. In such settings, stromal derived factor 1 (SDF1), angiopoietin 1 (Ang1) [16], [19] and other chemotactic factors are likely to diffuse from the injured area and influence NSC migration [20]. In the case of AD, NSCs modified to produce anti-amyloid biologics would be of greatest benefit if they could be induced to migrate to sites of amyloid deposition, or to distribute widely across the brain if they were modified to produce secreted agents.

Metalloproteinase 9 (MMP9) is up-regulated specifically by astrocytes reactive to Aβ deposits and genetic deletion of this protease in mice results in reduced catabolism of Aβ [21]. Neprilysin, endothelin-converting enzyme and insulin degrading enzyme are known to degrade Aβ, but in side-by-side comparisons with MMP9, only MMP9 degraded Aβ fibrils *in vitro* and Aβ plaques *in situ* [22]. Thus, MMP9 would appear to have potential to degrade pre-existing amyloid deposits.

In the present study, we sought to determine whether NSCs could be modified to secrete MMP9, and then tested whether transplanted NSCs expressing MMP9 could elevate levels of MMPs in the brains of two established mouse models of Alzheimer-type amyloidosis (APPswe/PS1dE9-Line 85 mice [23] and the tet-APPsi model [6]). We found that levels of endogenous MMPs were very high and that the enzyme delivered by NSCs had no obvious impact on the levels of amyloid deposits in either model. However, we did note that NSCs expressing MMP9 produced larger grafts; thus MMP9 may ultimately be a useful reagent for enhancing survival or migration of transplanted NSCs.

## Materials and Methods

### Isolation of NSCs

NSCs from the subependymal zone were isolated as previously described [10]. In brief, a rectangular forebrain block containing the subventricular zone was isolated from neonatal (P3-P8) B6 mice. The olfactory bulb, cerebellum, hippocampus, lateral portions of the striatum, and lateral and dorsal cerebral cortex were removed. The remaining tissue was minced with a razor blade, incubated in 0.25% trypsin/ethylenediaminetetraacetic acid (EDTA, Atlanta Biologicals, Lawrenceville, GA) and dissociated into a single cell suspension by triturating through a diametrically descending series of pipettes. Cells were then pelleted and washed several times before plating in NSC media. This media comprised of DMEM/F12 (Invitrogen, Carlsbad, CA) with 1× N2 supplements (Invitrogen, Carlsbad, CA), 5% fetal bovine serum, 100 U/mL penicillin, 100 µg/mL streptomycin, 35 µg/mL Bovine Pituitary Extract (Sigma, Carlsbad, CA), and 250 ng/mL amphotericin B (Fungizone; Invitrogen, Carlsbad, CA). NSC monolayers in T25 culture flasks were maintained in media supplemented with 20 ng epidermal growth factor (EGF) and 20 ng fibroblast growth factor (FGF; Sigma, St. Louis, MO) every two to three days [10]. Immediately prior to transplantation, monolayers were detached from culture flasks with 0.25% trypsin (Invitrogen, Carlsbad, CA), pelleted (1000× *g* for 5 min), washed twice with 200 µl of 1× Dulbecco's Phosphate Buffered Saline (dPBS) and diluted to approximately 5×10^4^ cells/µl in 1× dPBS. Two reference cell counts were performed on a hemacytometer to determine cell concentration.

### Animals

Experiments were performed on adult APPswe/PS1dE9 Line 85 mice, CamKII-tTa/tetAPPswe/ind (tetAPPsi) mice, and non-transgenic mice using protocols and procedures approved by the University of Florida Institutional Animal Care and Use Committee (Protocol #200801921). Line 85 mice constitutively overexpress chimeric mouse/human APP695 harboring the Swedish K670M/N671L mutations (Mo/HuAPPswe) and human PS1with the exon-9 deletion mutation (PS1dE9) [23]. In tet/APPsi mice, expression of APP can be suppressed by adding doxycycline, a tetracycline analog to the mouse chow [6]. These mice were raised without doxycycline to 10 months of age allowing plaque deposition to occur. Approximately two weeks prior to experimentation, a doxycycline diet was initiated to halt APP production. Both host mice and NSC donor mice were congenic on the C57BL/6J background, eliminating any problems with graft rejection.

### Surgical Procedures and Cell Transplantation

Mice were deeply anesthetized with 1–5% isoflurane. The top of the head was shaved and sterilized with alternating swipes of betadine antiseptic and 70% ethanol. The mice were securely mounted with ear bars and a nose bar to a stereotaxic apparatus (Kopf Instruments, Tujunga, CA). The anesthesia mixture was delivered through an inlet within the nose bar enclosure for the duration of the surgery. A sterile scalpel was used to make a small incision into the skin above the skull. The skin was reflected in order to expose Bregma and a hole was drilled to allow cells to be injected as described below. Following injection, the needle was left in place for 3 minutes to minimize backflow before being slowly withdrawn. The incision was closed with staples and mice were allowed to recover in a 37°C chamber.

In general, most of the infusion parameters used here are established protocols in other studies [18], [24], [25]. Cell numbers and volumes used here are similar to those used in intracranial surgeries by Park and colleagues [24], in which mouse brains were infused with 3×10^5^ NSCs in 8 µL. We took advantage of two infusion systems depending on experimental needs. The first system was an automated system for convection-enhanced delivery (CED), calibrated with positive pressure to deliver NSCs at 1 µL/min. CED results in nearly homogenous infusate concentration [26] and minimizes artifacts that may come about due to infusion pressure variability. Therefore, we used this system for short-term cell distribution studies. The system consisted of a 100 µL gas-tight syringe (Hamilton, Reno, NV) driven by a syringe pump (Cole-Parmer, Vernon Hills, IL) connected to polyaryletheretherketone (PEEK) tubing (ID = 0.381 mm, OD = 0.794 mm, length = 0.5 m, Upchurch Scientific, Oak Harbor, WA). The PEEK tubing was coupled to a silica cannula (ID = 50 µm, OD = 147 µm, Polymicro Technologies, Phoenix, AZ) via a microfluidic connector. We typically harvested 4–6 confluent T75 flasks per experiment and concentrated the cells at 5×10^4^ cells/µL in order to reliably have enough volume for transplantation and for void volume.

The second system we used was a Hamilton 33 gauge 10 µL syringe (Hamilton Company, Reno, NV). This system required manual manipulation but required less void volume. We were able to concentrate similar yields of NSCs into 1×10^5^ cells/µL and therefore infuse almost half as much volume in studies on the therapeutic potential of NSCs. Smaller volumes in these studies minimized risk of infusion-related damage to host tissue in long-term experiments. 0.25 µL was infused every 15 seconds.

In studies of NSC distribution, a single bolus of NSCs was infused into 8–14 month non-transgenic mice. We targeted NSCs to various brain subcompartments with coordinates away from Bregma in the anterior/posterior (AP), medial/lateral (ML), and dorsal/ventral (DV) planes. Cells were deposited bilaterally with coordinates targeting the fimbria (AP: −0.2 mm, ML: +/−1.2 mm, DV: −2.5 mm from the dura), thalamus (AP: −2.0 mm, ML: +/−1.5 mm, DV: −4 mm from the skull), striatum (AP: +1.18 mm, ML: +/−1.5 mm, DV: −4 mm from the skull) and hippocampus (AP: −2.00 mm, ML: +/−2.0 mm, DV:−1.5 mm, −2.1 mm, and −2.8 mm from the skull). Hippocampal DV coordinates correspond to the superior aspect, dorsoventral center, and inferior aspect of the septal hippocampus, respectively. ∼5×10^5^ cells (9.8 µL) were infused into each site of the thalamus, striatum and hippocampus; ∼1×10^5^ NSCs (1.96 µL) were infused into the fimbria to accommodate its smaller spatial dimensions.

In studies that focused on modifying amyloid pathology, we were interested in wide-spread distribution of NSCs in the hippocampus of 13–14 month old transgenic APPswe/PS1dE9 mice or 9–10 month old transgenic tetAPPsi mice. We therefore manually infused NSCs at 3 sites with the following coordinates: AP: +2 mm, ML: +/−2 mm, DV: 2.0 mm, −2.3 mm, and −2.5 mm from the skull. ∼1.25×10^5^ cells were deposited at −2.0 mm and −2.3 mm, while ∼2.5×10^5^ cells were deposited at −2.5 mm for a total of ∼5×10^5^ NSCs (4.9 µL). These coordinates targeted hippocampal gray matter in regions ranging from CA3, the molecular cell layer and the subgranular zone of the dentate gyrus.

Vehicle-only infusions are frequently used as controls in transplant studies. These are often performed on a parallel set of age-matched animals. In our experience, aged-matched APPswe/PS1dE9 mice have substantial variability in the amount of amyloid deposited. We addressed this concern by performing a cell-free, vehicle only, mock surgery in the equivalent area of the contralateral hemisphere of animals receiving NSCs. This control scheme allowed for direct comparison to each animals' individual amyloid burden while controlling for the immune response to endogenous cells killed during needle penetration.

### Preparation of Cells and Tissues for Analysis

To harvest tissues, mice were deeply anesthetized and euthanized by isoflurane overdose followed by immediate exsanguination and perfusion with cold 1× PBS. Whole brains were quickly dissected out and placed in cold 4% paraformaldehyde fixative overnight. Fixed brains were immersed in 30% sucrose before sectioning at 20 µm intervals within a cryostat. Sections were stored in anti-freeze media at −20°C until further processing. Cultures of NSC monolayers were similarly fixed and stored. Fixed tissue sections and NSC monolayers were blocked and permeabilized with 0.1% Triton-X, 10% goat serum in 1× PBS during immunochemical applications.

### Immunochemistry

Primary antibodies used in this study include anti-Aβ 6E10 (Signet, Dedham, MA), microglial antigen Iba1 (Wako, Richmond, VA), astrocyte antigen GFAP (Dako Corporation, Carpinteria, CA), pan neuronal antigen BIII-Tubulin (Covance, Princeton, NJ), anti-human MMP9 Clone 56-2A4 (Abcam, Cambridge, MA), and anti-mouse MMP9 (courtesy of R. Senior, Washington University). An antibody to copGFP (Evrogen, Moscow, Russia) was used to detect GFP if fluorescence was too low to detect directly. For both cultured cells and tissue sections, Alexa 488 and Alexa 568 goat secondary antibodies (Invitrogen, Carlsbad, CA) were used. Cell nuclei were stained with 4′,6-diamidino-2-phenylindole (DAPI, Invitrogen, Carlsbad, CA). Antibodies as supplied by the manufacturer were diluted from 1∶500 to 1∶2000 for use in immunohistochemistry. Immunostained cells and tissue sections were photographed with an Olympus DP71 camera mounted on an Olympus BX60 microscope or an Olympus DSU-IX81 Spinning Disc confocal microscope. Confocal images were deblurred by a nearest neighbor algorithm.

### Lentivirus Construction and Transduction

Self-inactivating Lentiviruses [27] were used to transduce NSCs with expression cassettes for plankton copepod green fluorescent protein (hereafter simply referred to as GFP; which is similar in size to EGFP (26 kDA) but has more fluorescence output [28]). Viral vectors were created using the pCDH cloning and expression system (SBI, Mountain View, CA). The pCDH vector contained cDNA for GFP under the elongation factor 1 (EF1) promoter. Human MMP9 (2.54 kB) cDNA within a pCMV6-XL4 plasmid vector (Origene, Rockville, MD) was cloned and then ligated into the pCDH Lentiviral vector using Not1 restriction sites. The Lentiviral vectors were packaged into Lentivirus pseudotyped with vesicular stomatitis virus G (VSV-G) glycoprotein using a three plasmid packaging system that included the pCDH construct (with or without the MMP9 transgene) as previously described [29]. Final viral titers were approximately 1×10^11^ particles/mL. Confluent cultures of NSCs were trypsinized and roughly 1×10^5^ cells were resuspended in 100 µL dPBS. These cells were transduced by exposure to 6 µL of virus concentrate for 1.5 hrs at 37°C, with agitation every 10–15 min. This ratio of virus to cells was found to most optimally and consistently yield NSCs expressing GFP. Transduced cells were grown to confluent cultures in T25 flasks. Cells were trypsinized and resuspended in 5 mls of dPBS with 2% fetal bovine serum. GFP expressing cells were then enriched by fluorescence activated cell sorting (FACS). NSCs with GFP intensity below an arbitrary threshold value of 10^3^ units were discarded. Cultures of remaining cells maintained high GFP expression up to 6 months following FACS enrichment. Real-time PCR was used to confirm conditioned media from expanded cultures contained no viral genomic DNA (data not shown) to ensure virus was not present in the injected NSCs cells.

### Quantification of NSC Transplant Dimensions

To determine whether tetramethylammonium (TMA) distributed anisotropically or isotropically, Mazel et al. quantified changes of TMA concentration along the orthogonal x, y and z axes [30]. We adapted this method to perform analyses on infused NSCs expressing GFP. An index of anisotropy, designated *A*, was determined by the quotient of x/y, where x represents the distance of engraftment on the transverse axis and y represents the vertical axis.
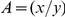
By definition, an ideal ellipsoid would have a larger value of *x* relative to *y* while a circle would have equal *x* and *y* values. In practice, *A* values close to 1 represent isotropy while large *A* values represent anisotropy. During sectioning, we punctured small holes into one hemisphere, so we could distinguish sides in bilateral infusions. For each infusion, one to two 20 µm sections containing engraftments were analyzed with AxioVision LE software (Zeiss, Jena, Germany). Images of samples with equal pixel/micrometer value were used to determine the distance of *x* and *y*. An unpaired, two-tailed Student's t-test using Microsoft Excel was used to compare *A* from different sites of infusion. A p-value of <0.05 was considered statistically significant.

To determine whether expression of MMP9 influenced the size of the graft, we used Image Pro Plus software (MediaCybernetics, Bethesda, MD). For this study, we focused on the area closest to the site of injection from each animal, looking for sections with evidence of needle track penetration. An unpaired, two-tailed Student's t-test using Microsoft Excel was used to compare values. A p-value of <0.05 was considered statistically significant.

### Image Segmentation and 3D Reconstruction of NSC Engraftments

A semi-automatic image segmentation routine was implemented in MATLAB (MATLAB 2010a, The MathWorks, Inc., Natick, Massachusetts USA) that distinguished regions of GFP expression from control regions of the brain by means of a threshold unique to each histological section. RGB images of the histological sections were imported into MATLAB; however, only the green channel of the images was used for image segmentation. The average background signal was determined by averaging the signal intensity in user-designated control regions of interest (GFP-free regions of tissue) for each section. Similarly, the standard deviation of noise was determined by evaluating the noise behavior in user-designated noise regions of interest (tissue-free regions in images). Thresholds were calculated by adding six times the standard deviation of noise to the control signal intensity for each section. The image segmentations were refined in ITK-SNAP [31], an open source medical image segmentation tool. Refinements included removing regions of auto-fluorescence (false-positives) from the image segmentation as well as segmentation of annotations such as scale bars. Three-dimensional reconstructions were generated by first aligning the histological sections prior to image segmentation and finally generating a mesh from the 3D image segmentation using ITK-SNAP's built in mesh generation tool.

### Semi-automated Analysis of Aβ Plaque Number in APPswe/PS1dE9 mice

Image Pro Plus software was used to quantify intensity over background for images of 6E10 immunoreactive Aβ plaques in coronal sections as previously described [32]. Objects that were <50 µm or had angular edges that did not correspond to Aβ plaques were excluded. Because transplanted cells largely settled in the corpus callosum, we focused on a region of interest (ROI) that included the dorsal hippocampus below the site of engraftment and the cortex above the site of engraftment (1.25 mm×2 mm). To be consistent across mice, the vertical and horizontal boundary of the ROI was the meeting of the dentate arms and the lateral blade of the dentate gyrus, respectively (see Fig. S2 for an example). For an internal control, analysis was performed on the equivalent region of the contralateral hemisphere (non-infused side). Ipsilateral versus contralateral plaque numbers were analyzed using paired, two-tailed Student's t-tests in Microsoft Excel. Plaque numbers in animals receiving MMP9-NSCs and GFP-NSCs were compared with an unpaired, two-tailed Student's t-test using Microsoft Excel. Similar analyses with NIH Image J were performed on MMP9-NSC unilateral infusions into tetAPPsi mice. However, in this cohort, a smaller ROI (0.25 mm×0.35 mm) was measured to better sample Aβ plaque burden in the immediate vicinity of engraftments; one edge of the ROI was aligned with the engraftment and the number of Aβ plaques was compared with the equivalent region in the contralateral hemisphere. A p-value of <0.05 was considered statistically significant.

### Aβ42 Degradation Assay

The method used to quantify the degradation of Aβ42 has been described previously [33]. Briefly, lyophilized Aβ42 peptide (rPeptide, Bogard, GA) was resuspended to 1 mg/mL in 1% NH_4_OH and stored at −20°C according to manufacturer's directions. Stock preparations contained monomeric (4 kDa), oligomeric (16 kDa, 20 kDa) and higher-order conformations larger than 220 kDa [33]. 1×10^5^ NSCs were seeded and allowed to grow within RS chambers (Nunc, Roskilde, Denmark) until confluent. Cultures were rinsed and given Aβ42 diluted in DMEM at 4 µg/mL. The cells were allowed to internalize Aβ42 from the media for 3 hrs in 37°C. The cells were then rinsed and incubated at 37°C with 1.5 mLs of culture media lacking Aβ42. This media and that of cells lysed immediately following rinsing were collected, as were the conditioned media and lysate from wells incubated for 3 hrs and 16 hrs. The lysis buffer based on NP40 (50 mM Tris, pH7.4, 250 mM NaCl, 5 mM EDTA, 50 mM NaF, 1 mM Na_3_V0_4_, 1% nonidet P40 [NP40] FNN0021, Invitrogen, Carlsbad, CA) was supplemented with 1× protease inhibitor cocktail (Sigma, St. Louis, MO) and 1 mM PMSF. To determine the fate of Aβ42, we employed a sandwich-style ELISA (Invitrogen, Carlsbad, CA) that is specific to full-length Aβ42 (capture antibodies have opposing N- and C-terminal affinities). ELISA readings of Aβ42 content in lysate allowed for quantification of NSC internalization and the subsequent loss of Aβ42 from cell lysates was a measure of degradation. Readings from the media assayed for the recycling of Aβ42 back to the media. The ELISA was processed with duplicates of each sample and absorbance read at 450 nm using a spectrophotometer (Bio-Tek, Winooski, VT). Data was normalized to culture plate wells that were treated with Aβ42, as described above, but contained no cells. This control was performed because some of the peptide adheres to the plastic plates non-specifically and is subsequently released by buffers that are used to lyse cells or leaches into the medium upon further incubation. The detection limit of the assay was 10 pg/mL. An unpaired, two-tailed Student's t-test using Microsoft Excel was used to compare values. A p-value of <0.05 was considered statistically significant.

### *In situ* Metalloproteinase Gelatinase Activity

To detect metalloproteinase activity *in situ*, we followed a protocol modified from Yan et al. [22]. Briefly, a 1% agarose solution with 1× PBS and 0.1% Triton-X100 was made homogeneous with three 10 sec pulses within a microwave oven followed by brief vortexing. DQ gelatin (100 µg/mL, Invitrogen, Carlsbad, CA) and DAPI (1∶1000) were then added to the agarose solution. A thin film of this mixture was quickly added to dry sections mounted on glass slides and allowed to set overnight at room temperature. Green fluorescence developed at sites where metalloproteinase digestion of DQ gelatin occurred. This reaction did not occur in sections from non-transgenic littermates and or with addition of 0.8 mM of the general metalloproteinase inhibitor 1,10 Phenanthroline (PNTL; Sigma, St. Louis, MO).

## Results

### Modification of NSCs to express MMP9

**Figure 1:**
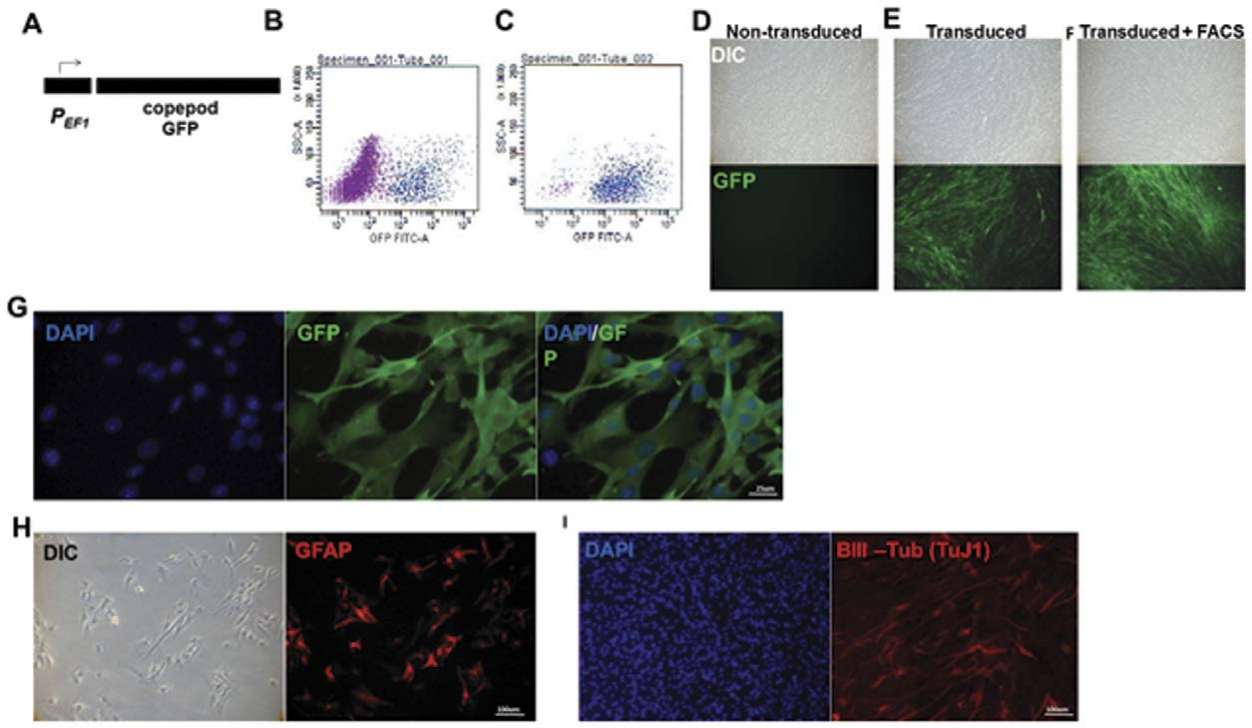
*Ex vivo* manipulation of NSCs. NSCs were transduced with Lentiviruses with GFP driven by the elongation factor 1 promoter (EF1) (A). Transduction involved exposure of NSCs to virus concentrate for 1.5 hours with intermittent agitation. Following transduction, a population of more fluorescent NSCs was observed by FACS analysis (B). Highly fluorescent cells defined by AU>10^3^ (C) formed cultures with more uniform and robust expression of GFP (D–G). Enriched NSCs formed expandable, long-term monolayers which expressed GFAP and BIII-Tubulin, markers commonly associated with multipotent astrocytic stem cells (H–I). GFP fluorescence was detected directly in B–F and indirectly with an antibody specific to GFP in G. The data are representative examples from three repeated isolations and transductions of NSCs.

Multipotent NSCs were derived from the subventricular zone of non-transgenic neonatal mice and cultured for multiple generations as we have previously described [10]. Self-inactivating Lentiviruses [27] were used to generate NSCs expressing GFP (GFP-NSCs) (Fig. 1A). Upon Lentiviral transduction, a subpopulation of GFP-NSCs showed GFP fluorescence within three days and remained fluorescent for up to six months. Fluorescence Activated Cell Sorting (FACS) technology was used to enrich undifferentiated GFP positive NSCs (Fig. 1B–G). These cells formed expandable monolayers that were immunoreactive to BIII-Tubulin, a pan neuronal marker recently associated with subventricular zone neuronal precursor chains [10]; GFAP, an astrocyte marker; and to a lesser extent, the microglial marker, Iba1 (Fig. 1H–I; Fig. S1). BIII-Tubulin and GFAP are markers previously associated with NSCs [10], [34]. We have previously shown that microglia are found in NSC cultures and are thought to produce factors necessary for neurogenesis in NSC cultures [35].

**Figure 2:**
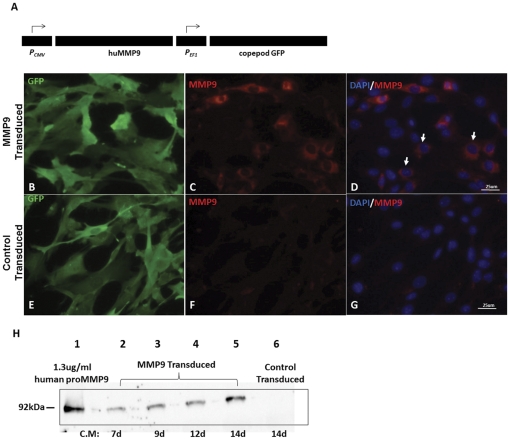
Expression and secretion of MMP9 in cultured NSCs. Cells were transduced with Lentiviruses that confer cytomegalovirus (CMV) directed human MMP9 expression (A). MMP9 immunoreactivity was robustly detected in cells transduced with the MMP9 virus (B–D). Arrow indicates MMP9 expressing cells with little detection in cells transduced with GFP only virus (E–G). Immunoblots of medium from cultured cells demonstrated secretion of human MMP9 into the medium (H). Each lane contains 18 µL of conditioned medium from cells incubated for 7–14 days. Lane 1 was loaded with 18 µL of a 1.3 µg/mL solution of purified human MMP9 proenzyme.

To develop NSCs as delivery vehicles for biologic therapeutics, we tested whether these cells could be engineered to express MMP9. For reasons delineated above, secreted MMP9 is an excellent candidate biologic for enhancing the clearance of amyloid deposits. Lentiviruses were thus constructed to co-express human MMP9 and GFP for transduction of NSCs (Fig. 2A). Following procedures similar to those outlined above, we enriched for cells expressing high levels of GFP by FACS and ultimately produced NSC cultures with a similar level of GFP expression as control NSCs expressing only GFP (Fig. 2B and E). Immunostaining of these cultures with antibodies to MMP9 demonstrated that only the cells transduced with MMP9 Lentiviruses expressed the enzyme, which appeared to be located in the endoplasmic reticulum (Fig. 2C, D compared to F, G; the strongest immunoreactivity was concentrated around the nucleus). To determine whether the enzyme was secreted, culture medium was harvested from cells incubated for 7–14 days (Fig. 2H). The enzyme was readily detected in culture medium with a relative molecular mass of a size expected for the proenzyme. Together, these studies demonstrate the relative ease with which NSCs can be modified to express and secrete potentially therapeutic molecules.

### Distribution of Transplanted NSCs

**Figure 3:**
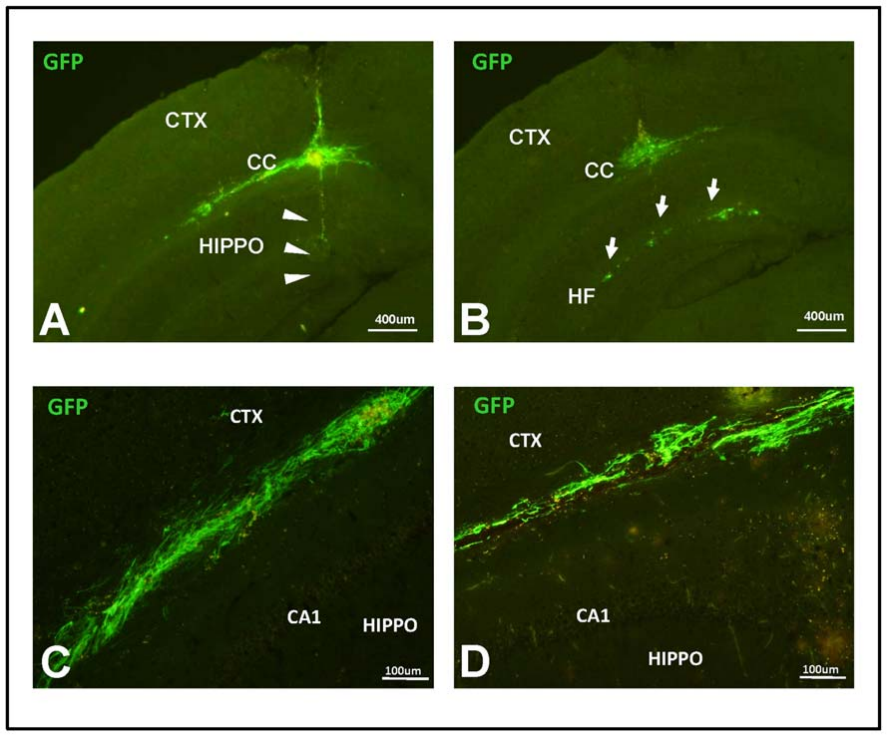
Distribution of long-term NSC engraftments in APPswe/PS1dE9 mice. Symptomatic APPswe/PS1dE9 mice received NSCs at three depths per infusion and were allowed to survive for one month. NSCs were mainly distributed in the corpus callosum despite evidence of needle track penetration into the hippocampal gray matter (A; arrowheads indicate approximate position NSCs were released). In some instances, NSCs formed secondary grafts in the hippocampal fissure (B; arrows). Closer inspection in NSCs engrafted in the corpus callosum of APPswe/PS1dE9 mice (C) and tetAPPsi mice (D) revealed these cells had an elongated morphology characteristic of migrating cells; however, cells did not exit into the cortex (CTX) or spread below CA1 neurons of the hippocampus (HIPPO). GFP expression was detected indirectly with an antibody specific to GFP in all panels.

NSCs expressing either MMP9 with GFP or GFP alone were infused into the hippocampus of 13–14 month old APPswe/PS1dE9 mice. Mice were subsequently harvested one month later to assess the distribution of the transplanted cells. Despite evidence of needle track penetration into the hippocampal gray mater (Fig. 3A), the majority of engrafted cells were located in the overlying corpus callosum (Fig. 3A–C, n>9 animals). A secondary site of engraftment in 33% of the animals was the hippocampal fissure (Fig. 3B). For our preclinical assessments, we also used the tetAPPsi mouse model (see Methods). A similar distribution of NSCs was observed in these mice (Fig. 3D). Thus, although the intended target was the hippocampus, these NSCs tended to colonize the overlying white matter tracts.

### Therapeutic effects of MMP9-NSCs on amyloid deposition

**Figure 4:**
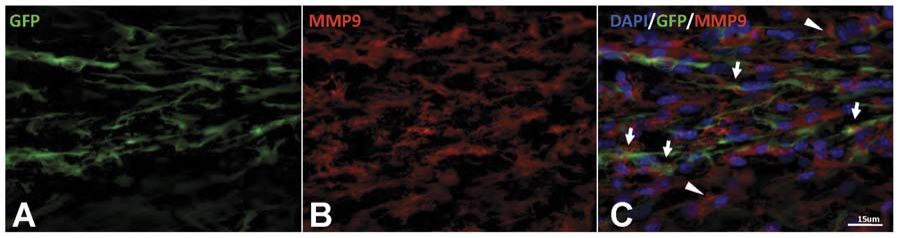
*In vivo* expression of MMP9 by transplanted NSC cells. Confocal image of engraftment shows NSCs (arrows) and host cells (arrowheads) were immunoreactive for MMP9 (A–C). The images shown are representative of an analysis of three sections (each) from three animals. 1 µm z-plane depth in A–C.

For our first test of whether NSCs could deliver MMP9 to the CNS at effective levels, MMP9-NSCs and NSCs expressing only GFP were transplanted into the hippocampus of 13–14 month old APPswe/PS1dE9 mice. Mice were subsequently harvested one month later. With immunohistochemical stains for MMP9, we were able to identify cells within the graft area that expressed MMP9 (Fig. 4 A–C). However, immunoreactivity was not confined to the area of the graft as cells adjacent to the NSCs, marked by GFP expression, also immunostained for MMP9. It is possible that there was cross-reactivity to endogenously expressed MMP9. We note that very little MMP9 is normally expressed in mouse brain, but what little mRNA that is expressed is present in the white matter tracts of the corpus callosum (see Allen Brain Atlas; www.mouse.brain-map.org). Thus, the immunoreactivity to MMP9 antibodies that was seen adjacent to the grafts is likely to be endogenous MMP9.

**Figure 5:**
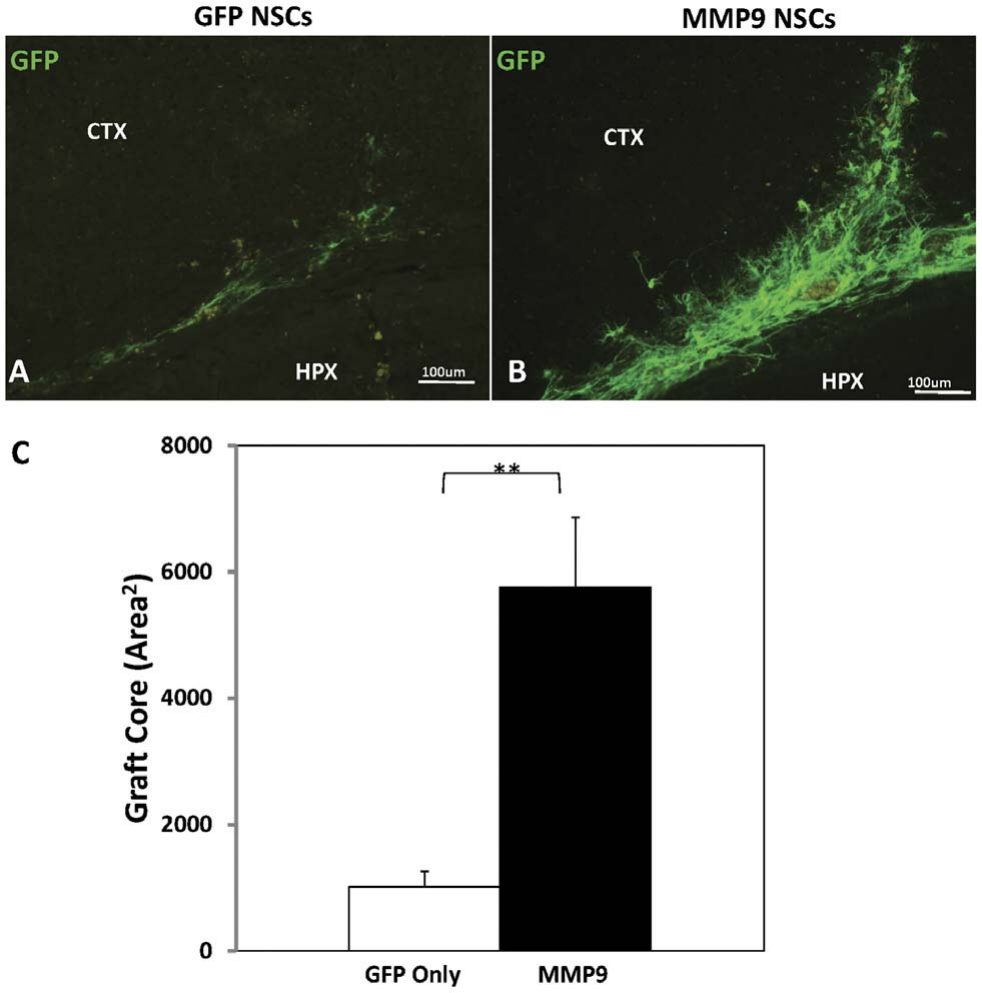
NSC engraftment is enhanced by MMP9 overexpression. MMP9-NSC grafts (n = 5) were larger than GFP-NSC grafts (n = 4) in APPswe/PS1dE9 mice (A–B). Aggregate data on the area of GFP immunoreactivity in sections with evidence of needle track penetration indicated grafts were 82.4% larger for MMP9-NSCs compared to those expressing only GFP (C). **p<0.01.

One of the most obvious effects of MMP9 expression was noted by the size of the grafts formed by NSCs expressing MMP9; MMP9-NSCs formed grafts that were 82.4% larger than grafts formed by NSCs expressing only GFP (Fig. 5, p<0.01). Thus, either more of the MMP9-NSCs survived injection or these cells migrated further away from the injection site.

**Figure 6:**
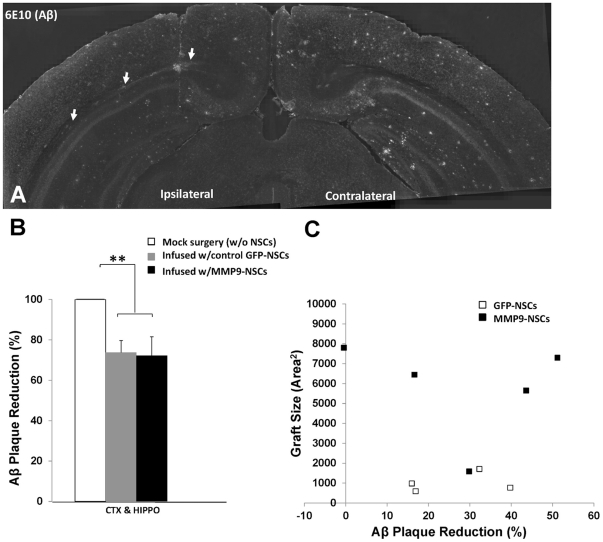
NSC transplantation but not MMP9 overexpression was associated with reductions in Aβ plaque burden in APPswe/PS1dE9 mice. In mice with month-long engraftments, Aβ plaques were reduced in the area proximal to grafts in the hemisphere receiving NSCs (A, ipsilateral hemisphere, arrows mark the location of the graft). Across all animals, Aβ plaques were reduced by 27% (B, n = 9 animals). Mice receiving MMP9-NSCs (n = 5) had substantially enlarged engraftments relative to GFP-NSCs (n = 4). However, the magnitude of Aβ plaque reduction was similar in both cohorts (B–C, closed squares = MMP9-NSCs, open squares = GFP-NSCs) and there was no obvious relationship between the size of the graft and the magnitude of plaque reduction. Aβ plaque burden determined from five to seven sections per animal. Engraftment size in each animal was determined in sections containing a needle track (**, p<0.01).

To determine whether transplantation had any effect on plaque burden, we sampled the amount of Aβ plaques around the engraftments (see Fig. S2 for an example of how regions of interest were outlined; ROI = 1.25 mm×2 mm). Because the rate of amyloid deposition varies between animals, we focused our comparison to plaque burden in the contralateral, mock-infused hemisphere as an internal control. Additionally, we compared animals that received MMP9-NSCs to animals receiving NSCs that expressed only GFP. Most animals exhibited reductions in plaque burden with some displaying notably strong effects (Fig. 6A, C). In total, the amount of plaque reduction in all mice was a modest 27% (Fig. 6B, n = 9, p<0.01). Animals hosting MMP9-NSCs had 28.6% less Aβ plaques (n = 5, p = 0.03) and animals hosting NSCs expressing only GFP had 26.4% less Aβ plaques (Fig. 6B; n = 4, p = 0.04). The difference in plaque reduction between animals with NSCs engineered to overexpress MMP9 and animals with NSCs expressing only GFP was not statistically significant. Thus, it appeared that transplantation of NSCs lowered plaque numbers in the area proximal the graft. A similar outcome was observed when we examined tetAPPsi mice (+ Doxycylcine) that had been unilaterally injected with NSCs expressing MMP9 and allowed to survive for 2 months (Fig. S3A–B). Compared to the same location in the contralateral side, the area near NSC grafts on the injected side seemed to contain less amyloid plaques (26% less, n = 7). However, in a second set of tetAPPsi mice (+Doxycycline) we infused NSCs expressing MMP9 in one hemisphere and NSCs expressing only GFP in the contralateral hemisphere to allow a direct side-by-side test of MMP9 efficacy on pre-existing Aβ plaques. Following a month of engraftment, no obvious differences in plaque burden were observed (see Fig. S3C for a representative example, n = 9). We therefore conclude that NSC-delivered MMP9 had no profound therapeutic effect on plaque burden; though, as observed above, the mere presence of NSCs produced a small but measurable reduction in plaque numbers in regions of the brain proximal to the graft.

Although we could find no evidence that MMP9 produced by transplanted NSC cells could lower amyloid plaque burden, we were intrigued by the observation that the area proximal to NSCs seemed to have less amyloid plaques. This effect was noted in the APPswe/PS1dE9 mice and in the tet-APPsi (+Doxycyline). This observation suggested that NSCs might have some inherent ability to lower Aβ levels and slow the rate of amyloid deposition. Using methodology we previously used to quantify microglial Aβ42 catabolism [33], we determined that cultured NSCs internalized and degraded Aβ42 added to culture medium (Fig. S4A–B). Thus, one possible explanation for the lower amyloid plaque burden near NSC grafts could be due to increased catabolism of Aβ peptide that slows the rate of amyloid deposition. However, we noted that the size of the graft showed no correlation with the degree of amyloid plaque reduction (Fig. 6C). In general, the larger grafts appeared to have more NSCs distributed over a wider area and grafts formed by NSCs expressing MMP9 were uniformly larger. Yet, there was no obvious correlation between graft size and proximal amyloid plaque numbers. Thus, it is difficult to conclude that the reductions in amyloid were a direct consequence of some activity by transplanted NSCs.

### High levels of metalloproteinase activity adjacent to dense cored amyloid deposits

**Figure 7:**
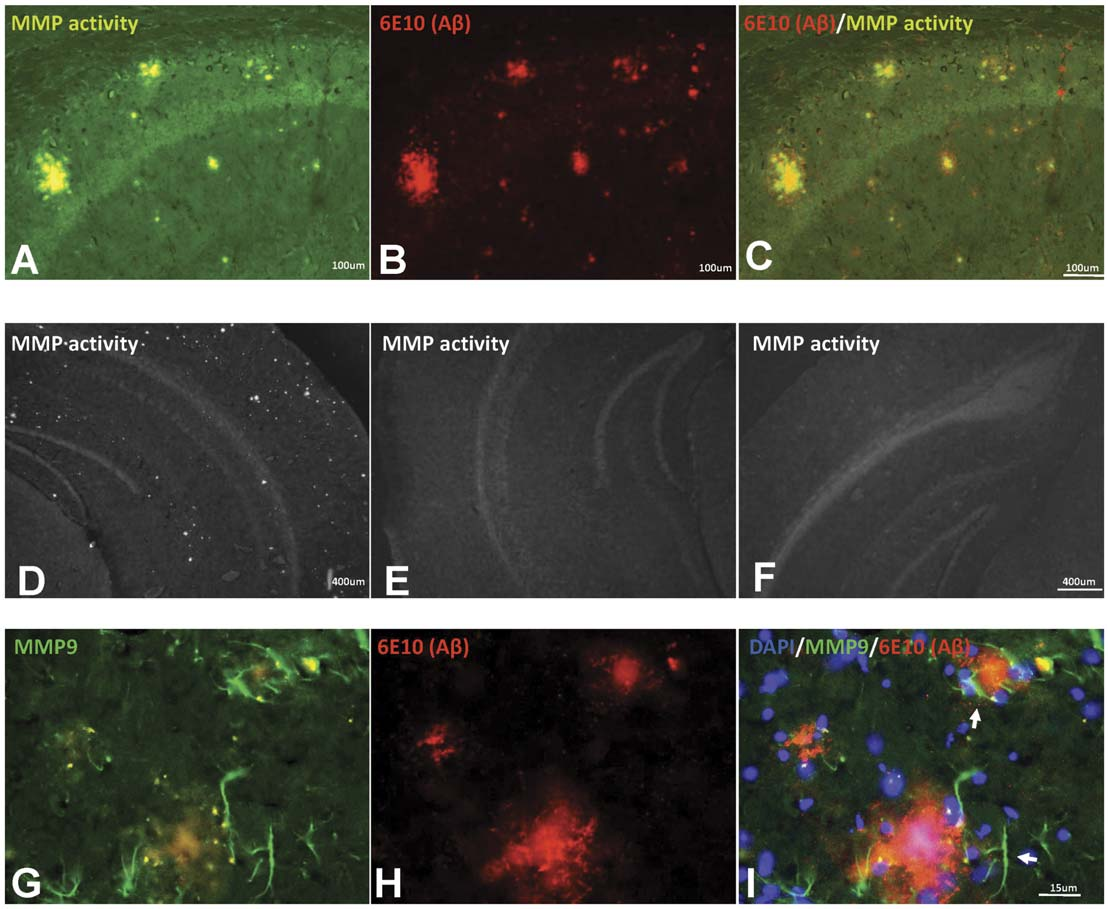
Localized endogenous MMP activity around Aβ plaques. A thin film of DQ gelatin was digested by metalloproteases, releasing fluorescence. This degradative activity occurred mainly around Aβ plaques (A–C). The activity of metalloproteases continued despite no new deposition of Aβ for one month in tetAPPsi mice (D). MMP activity was not observed in non-transgenic littermates (E) or in sections treated with the metalloprotease inhibitor, 1,10 PNTL (F). Antibody staining specific for mouse MMP9 indicated reactive endogenous cells (arrows) in close proximity to Aβ plaques deliver MMP9 (G–I) in APPswe/PS1dE9 mice. The images shown are representative of an analysis of at least three sections from three animals.

To determine whether transplantation of MMP9-NSCs induced greater metalloprotease activity around Aβ plaques, we employed an *in situ* metalloproteinase activity technique in which tissue sections were overlaid with agar containing a substrate that is selectively cleaved by metalloproteinases (see Methods). A previous study that used this technique described metalloproteinase activity adjacent to Aβ plaques that were surrounded by astrocytes with up-regulated MMP9 immunoreactivity [22]. We observed a similar concentration of MMP activity around amyloid deposits with no obvious increase in metalloproteinase activity levels around Aβ plaques in mice receiving MMP9-NSCs transplants. In performing these assessments, we were impressed by the high levels of metalloproteinase activity around Aβ plaques in transgenic mice (Fig. 7). Indeed the accuracy with which metalloproteinase activity derived fluorescence identified Aβ plaques was similar to that of immunostaining for Aβ (Fig. 7A–C). Virtually every plaque showed intense fluorescence indicative of high metalloproteinase activity. Robust MMP9 activity around amyloid plaques was also observed in tetAPPsi mice in which the expression of mutant APP, and thus the production of new Aβ deposition, had been halted for a month (Fig. 7D). MMP activity was not observed in non-transgenic littermates (Fig. 7E) or in sections treated with the metalloprotease inhibitor, 1,10 PNTL (Fig. 7F). As previously reported, cells with up-regulated MMP9 were found in close proximity to Aβ plaques (Fig. 7G–I) [22]. These findings indicate that there are high levels of endogenous MMP activity associated with amyloid plaque deposits and it is thus difficult to predict how much additional MMP activity must be delivered to augment this natural activity, or whether higher levels of activity would be efficacious.

### Factors that affect the distribution of transplanted NSCs

**Figure 8:**
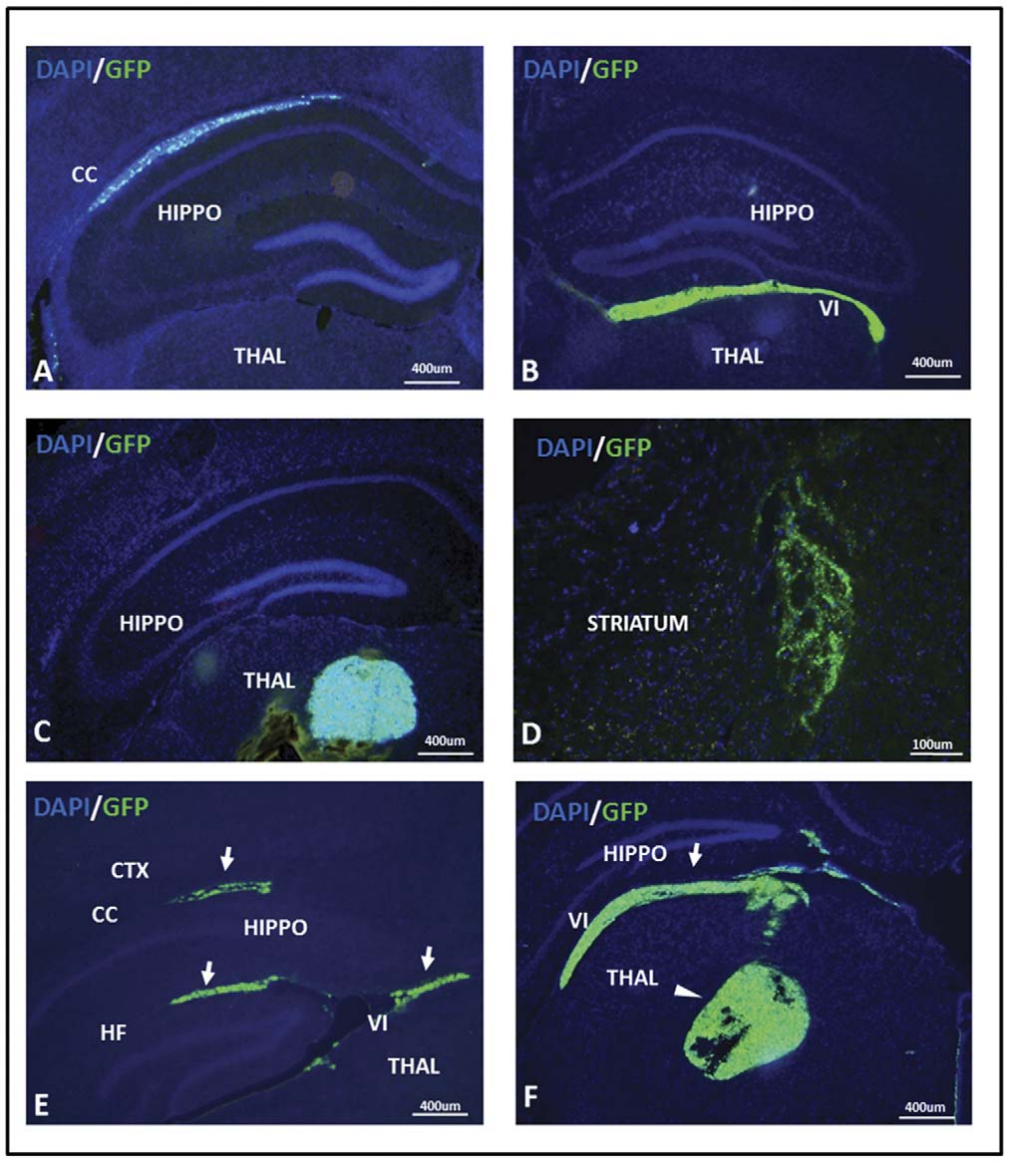
Short-term engraftments demonstrate the hippocampus specifically features anisotropic transport. NSCs were surgically infused into the brains of non-transgenic mice that were then sacrificed within fifteen minutes after cessation of infusion to observe near-immediate transplant distribution. NSCs infused proximal to the superior aspect of the septal hippocampus (HIPPO) were mainly distributed along the transverse plane of the corpus callosum (CC) (A). Similarly, NSCs distributed along the transverse plane of the velum interpositum (VI) when deposited more ventrally in the inferior aspect of the hippocampus (B). Engraftments in the thalamus (THAL) (C) and striatum (D) did not preferentially distribute in the transverse plane. 38.5% of infusions resulted in independent anisotropic (ellipsoid; arrows) and isotropic (circular; arrowheads) engraftments distal to the target site including the hippocampal fissure (HF) (E–F). GFP fluorescence was detected directly in all panels. The images shown are representative of an analysis of at least three tissues sections (each) from a minimum of three infusions.

In all animals examined, the primary location of engrafted NSCs, whether expressing MMP9 with GFP or GFP alone, was the corpus callosum and the hippocampal fissure. This pattern of distribution is highly similar to anisotropic distributions observed previously with small molecule infusions [26], [30], [36]. We therefore repeated NSC infusions in non-transgenic mice to determine whether the engraftment of NSCs shared spatial and temporal properties similar to small molecules. In these experiments, NSCs were bilaterally infused into the hippocampus and host mice were sacrificed within fifteen minutes of the cessation of infusion. We also infused NSCs into thalamus and the striatum to determine whether engraftment patterns in the hippocampus are unique. Across various depths of infusion of NSCs into the hippocampus, we consistently observed well-defined ellipsoid distributions of GFP fluorescence in the transverse plane (Fig. 8A–B, E–F) similar to engraftments in AD mice and previous reports of small molecule infusion. GFP fluorescence was found distributed largely in three structures: the corpus callosum, the velum interpositum and the hippocampal fissure. Specifically, NSCs that were infused into the superior aspect of the septal hippocampus yielded GFP fluorescence distributions throughout the corpus callosum (Fig. 8A; n = 5). NSCs targeted to the inferior aspect of the septal hippocampus produced GFP fluorescence distributed mediolaterally along the velum interpositum (Fig. 8B; n = 7). The velum interpositum is a soft tissue partition between the diencephalon and the telencephalon rather than a cavity or space [37]. Functionally, the velum interpositum forms the roof of the 3^rd^ ventricle and connects the choroid plexus of this ventricle to that of the lateral ventricle. The distribution of GFP fluorescence extended hundreds of micrometers along the velum interpositum, from the 3^rd^ ventricle to lateral regions such as above the dorsal lateral geniculate nucleus. On the other hand, NSCs that were infused into the thalamus (Fig. 8C; n = 7) and striatum (Fig. 8D; n = 3) produced spherical distributions of GFP fluorescence that were indicative of isotropic transport. In some instances, GFP fluorescence was not only at the position the cells were targeted, but also hundreds of micrometers away (Fig. 8E–F). 3D reconstruction of serial sections of a representative corpus callosum infusion illustrated the sheet-like spread of GFP fluorescence across the wide, flat bundle of corpus colossal fibers (Fig. S5A). In contrast, thalamic infusion reconstruction shows a globular spread of GFP fluorescence (Fig. S5B). These patterns of distribution support the idea that cells are distributed along anisotropic pathways at the time of injection (Fig. S5C). Importantly, the contours of transverse engraftments, whether in mice observed within minutes or a month after surgery, were nearly identical (Fig. S5D). Furthermore, NSC injections in non-transgenic mice resulted in cells that initially colonized white matter tracts (such as the fimbria) and moved laterally within this structure, but did not migrate into adjacent gray matter (Fig. S6).

## Discussion

The properties of NSCs, namely long-term *in vitro* expansion and engraftment potential [10], [34], have generated significant interest for application towards novel therapies such as cell replacement and therapeutic delivery. In this study, we examined NSCs as a delivery vector for secreted MMP9, a candidate AD therapeutic. Our methods of delivery resulted in NSC engraftment primarily white matter tracts with a location of the grafts sharing spatial and temporal characteristics with anisotropic distributions of small molecules. We found no evidence for significant migration of the NSCs into surrounding gray matter, even in animals that had survived for more than a year after transplantation. The primary effect of expressing MMP9 in these studies was to increase the size of the graft formed by the transplanted cells. We found no evidence that NSC-delivered MMP9 produced any effect on amyloid burden. Through the course of analyzing the effect of MMP9-expressing NSCs on amyloid plaque burden in our two models of Alzheimer-type amyloidosis, we found robust endogenous metalloproteinase activity specifically around Aβ plaques. Notably, this natural response failed to clear plaques even in the brains of the tet-APPsi mice in which expression of mutant APP had been inhibited by doxycycline. One may interpret this to mean that metalloproteases, even in favorable conditions in which deposition of new amyloid has been interrupted, are not particularly effective in clearing amyloid deposits.

### Inherent therapeutic value of NSCs as anti-amyloidogenics

One potentially interesting observation was that the area near the engrafted NSCs, whether expressing MMP9 and GFP or GFP alone, showed modest reductions in amyloid plaque numbers in the APPswe/PS1dE9 model and in tetAPPsi mice. We also noted that unmodified NSCs in culture showed the ability to degrade Aβ peptides. One interpretation of these observations is that NSCs may possess some ability to degrade Aβ and thus slow the formation of new amyloid plaques and/or induce clearance of pre-existing deposits. However, as mentioned above, the major effect of expressing MMP9 was to increase the size of the graft and we noted no linkage between graft size and amyloid load reduction. The larger grafts appeared to contain more NSCs, and thus we would have expected a greater effect with a larger graft size. Therefore, we are not inclined to conclude that the transplanted NSCs had any effect on amyloid burden. We note that a previous study by Blurton-Jones and colleagues in which NSCs were injected into hippocampus failed to detect any local effects on amyloid deposition [13]. Thus it is likely that the local reduction in amyloid plaque numbers we observed may be due to some secondary effect of transplantation.

### Potential efficacy of MMP9 delivered by NSCs in amyloid clearance

Our primary focus was to determine whether NSCs could be used to deliver MMP9 to hippocampal regions of the brain to catalyze reduction in amyloid burden. Despite evidence of good expression of MMP9 by these cells in culture, this approach did not result in significant reductions in Aβ burden as compared to the effects of transplanting NSCs expressing only GFP. Because of the nature of negative data, we cannot definitively conclude from our studies that NSC-mediated delivery of MMP9 will have no benefit in humans. However, elaborating on possible reasons for the lack of positive MMP9 effects may inform future studies on metalloproteinase therapy. The potential reasons for lack of efficacy mainly fall into three categories: the efficacy of metalloproteinases towards plaque reduction, the dose and activity of MMP9 delivered by NSCs, and finally, the proximity of NSC-delivered MMP9 to sites of amyloid deposition.

A discouraging finding regarding the potential efficacy of MMP9 was that we observed high levels of metalloproteinase activity around Aβ plaques in APPswe/PS1dE9 and tetAPPsi mice at baseline. Indeed, *in situ* assay for metalloproteinase activity on tissue sections provided a very specific and robust marker for Aβ plaques. We also observed high levels of metalloproteinase activity in tetAPPsi mice during treatment with doxycycline, indicating that Aβ deposits persist in this model despite high levels of metalloproteinase activity in proximity to Aβ plaques. Yan et al. previously noted metalloproteinase activity in proximity to amyloid deposits in another strain of mice that model Alzheimer-type amyloidosis [22], but interpreted the finding as evidence that MMP9 may be acting as an endogenous mechanism of clearance. In light of studies of mice that inducibly express mutant APP in which it has been demonstrated that Aβ plaques are very slow to spontaneously clear [6], it is difficult to imagine that metalloproteinase can readily clear densely packed amyloid deposits.

However, an important consideration in assessing the potential efficacy of MMP9 in clearing amyloid deposits is that it is very difficult to know what concentration of enzyme would be required to clear senile plaques. *In situ* experiments by Yan and colleagues suggested 70 nM (6.4 µg/mL) is sufficient to degrade Aβ plaques [22]. We estimate that conditioned media from MMP9-NSCs contains 3 µg/mL of MMP9. Because the volume of the medium would be much greater than the volume of the interstitial space around the engrafted NSCs, the host tissue around NSC engraftments may have contained MMP9 at concentrations approaching the dose found effective *in situ*. However, we do not know what fraction of the secreted proMMP9 enzyme was fully activated, how quickly it may have been cleared, or whether it diffused far enough from the graft site to reach the target amyloid plaques.

### NSC transplantation in therapeutic applications

One lesson learned from these studies is that there remain fundamental questions regarding how engrafted NSCs are ultimately distributed after transplantation. Cell migration is clearly one determinant of graft distribution. For instance, we have previously shown that SVC NSCs transplanted into the lateral ventricle follow migrational cues in the rostral migrational stream [10]. Consequently, we expected at least some NSCs to follow directional cues in the subgranular zone, in the ventricular walls, or respond to chemoattractants that draw astrocytes to Aβ plaques. The fact that these possibilities were not observed suggested factors other than migration may have determined graft localization.

To gain insight on how NSCs became distributed, we examined NSCs immediately after injection and after long-term engraftment. Unexpectedly, we found that host mice harvested only minutes after surgery or after a month had remarkably similar distributions of NSCs. These distributions were stereotypically on the transverse plane and occurred mainly in the corpus callosum, but were also observed in the hippocampal fissure and the velum interpositum. Infusates of small molecules and macromolecules also occur within minutes, are stereotypically in the transverse plane, and are specific to structures such as the corpus callosum, but not hippocampal gray matter [26], [30], [36]. These spatio-temporal similarities suggest factors that determined the distribution of small molecules also influenced NSC distribution in our transplant studies.

Interestingly, we noted that MMP9-NSC engraftments were 82.4% larger than those of GFP-NSCs. The enlargement of engraftments due to MMP9 overexpression has also been observed in muscle stem cell transplants [38]. These investigators interpreted the larger engraftments as evidence that MMP9 expression induces migration. Whether the larger grafts formed by MMP9-expressing NSCs was a consequence of greater migration with the white matter tracts or greater survival of the engrafted cells remains to be determined. However, this finding raises the possibility that expression of MMP9 could have utility in therapeutic applications of NSCs as a means to enhance survival or migration.

Finally, the morphology of the engrafted NSCs in this study was similar to that of immature cells in the white matter tract of the rostral migratory stream seen in our previous work [10]. Our transplants were GFAP immunoreactive (data not shown) and it is unlikely that these cells underwent significant differentiation [39]. Since the main criterion for their usefulness in the current study was the expression of potentially therapeutic proteins over long periods of time, the terminal differentiation state of the transplanted cells was not a primary concern. In other applications of NSC transplantation, the terminal differentiation state may well be a major factor in efficacy.

### Conclusions

In summary, we have performed a preclinical assessment of the ease with which NSCs can be manipulated to express biologic therapeutics, and whether such cells can form significant engraftments in the brains of mice that exhibit Alzheimer-type amyloid pathology. In this test case, we investigated the potential efficacy of MMP9 in promoting the clearance of amyloid deposits. Although we demonstrate that NSCs can indeed be easily modified to express MMP9 and that NSCs can form significant grafts that survive for long periods, we observed no indication that the cells delivered a sufficient quantity of enzyme to be efficacious. A confounding factor in the study was the high level of endogenous MMP activity associated with amyloid plaques in these mouse models. This result leads us to question whether raising MMP9 levels would have any practical benefit, what level of activity would be required to be efficacious, and whether NSC-mediated delivery could raise the levels high enough to be of benefit. Although our results with MMP9 are not encouraging in the context of AD, there are clearly a multitude of applications in which NSC transplantation could be used in therapeutic applications as delivery vehicles or as supportive replacement cells. Moreover, the ability of these cells to survive long-term in the brains of animals with significant disease pathology provides encouragement for further development of this approach.

## Supporting Information

Figure S1 **NSC monolayers contain a subpopulation of cells reactive to antibodies against the microglial marker, Iba1.** The images shown are representative of an analysis of three experiments.
(TIF)

Figure S2 **Quantification of Aβ plaque burden in APPswe/PS1dE9 mice.** A defined area of consistent size (ROI: 1.25 mm×2 mm) was outlined on each hemisphere, encompassing part of the cortex and hippocampus. 6E10 immunoreactive Aβ plaques (arrowheads) within this region were counted and compared.
(TIF)

Figure S3 **Graft associated reduction of Aβ plaques in tetAPPsi mice.** TetAPPsi mice on doxycycline diet to inhibit new Aβ deposition hosted MMP9-NSCs in one hemisphere for two months. Transplantation was associated with less plaques in all but one animal (A, n = 7). Across all mice, grafts were associated with 26.1% less Aβ plaques (B). The number of Aβ plaques in a graft-associated ROI of 0.25 mm×0.35 mm were counted with NIH Image J (see Methods). In follow-up experiments, GFP-NSCs and MMP9-NSCs were bilaterally engrafted for a month. No obvious differences were observed in this side-by-side test on the effect of NSC derived MMP9 on pre-existing Aβ deposits (C, representative image from n = 9, arrows mark the position of the grafts).
(TIF)

Figure S4 **NSCs possess an inherent capacity to degrade Aβ42 peptides.** Cultured NSCs were bathed in media containing 4 µg/mL of Aβ42 (n = 4) before we subsequently assayed Aβ42 levels in cell lysates and culture medium at 3 and 16 hours later by ELISA. The medium was analyzed to determine whether any peptide taken up by these cells was recycled back to medium. In all cases we included a sample in which no cells were present in the culture vessel to control for non-specific binding of Aβ to plastic. Note that there is significant nonspecific adherence of the peptide to plastic, and significant re-solubilization occurs when medium is reapplied to the vessel. Despite the unavoidable problems with non-specific binding of Aβ to culture vessels, we observed a very significant increase in Aβ levels in cell lysates after incubation with levels quickly falling back to baseline levels (A). As Aβ levels in cell lysates declined, we did not observe a parallel increase in Aβ levels in medium above what occurs from leaching from the plastic (B 3 hrs, *p*<0.01; 16 hrs, *p*<0.01). Therefore, we conclude that NSCs were degrading the internalized peptides. Statistical analyses were performed as described in Materials and Methods: *,*p*<0.05; **, *p*<0.01.
(TIF)

Figure S5 **Geometric analyses of short-term NSC engraftments.** 3D reconstruction of serial sections shows engraftments form a sheet-like spread in the corpus callosum (A) and a globular spread in the thalamus (B). To distinguish these differences numerically, the travel distance of NSCs in the transverse plane (*x*) was divided by the travel distance in the vertical plane (*y*), yielding an anisotropic value, *A. A* is 7.3× larger in the corpus callosum compared to the thalamus (C). Similar comparison indicates *A* values of the hippocampal fissure and velum interpositum are 3.7× and 6.8×, respectively, to the thalamus (**, p<0.01, comparison to thalamus). Comparison of the index of anisotropy of long-term engraftments in the corpus callosum to short-term engraftments in the corpus callosum and velum interpositum reveals near identical values (D), indicating month-long engraftments in AD mice are anisotropic. The anisotropic values were calculated from an analysis of a minimum of three infusions for each structure indicated. Anisotropic values for long-term engraftments were calculated from APPswe/PS1dE9 mice.
(TIF)

Figure S6 **Engraftment in white matter results in permanent localization of NSCs in white matter.** We infused NSCs into the fimbria, a white matter structure along the medial edge of the hippocampus. NSCs infused into the fimbria are positioned to migrate into the hippocampus, the thalamus or enter the lateral ventricle. Non-transgenic mice were used to avoid the possibility of effects specific only to AD pathology. Observation of NSCs shortly after infusion (<15 min) revealed graft cores of cells in close association of each other within the fimbria (A, n = 6). On the other hand, cells that were engrafted for one year migrated within the fimbria to the walls of the lateral ventricle and near CA3 (B–C, n = 3). No cells were found in the hippocampus or the thalamus (C, arrowheads indicate migrating cells, dashes mark hippocampal border). Thus in our models, NSCs migrated within white matter but did not exit to surrounding structures.
(TIF)
