# Supplementary figures and images for: A Preclinical Assessment of Neural Stem Cells as Delivery Vehicles for Anti-Amyloid Therapeutics
Source: PLoS One. 2012 Apr 4;7(4):e34097. doi: 10.1371/journal.pone.0034097 (PMC3319561; doi:10.1371/journal.pone.0034097)

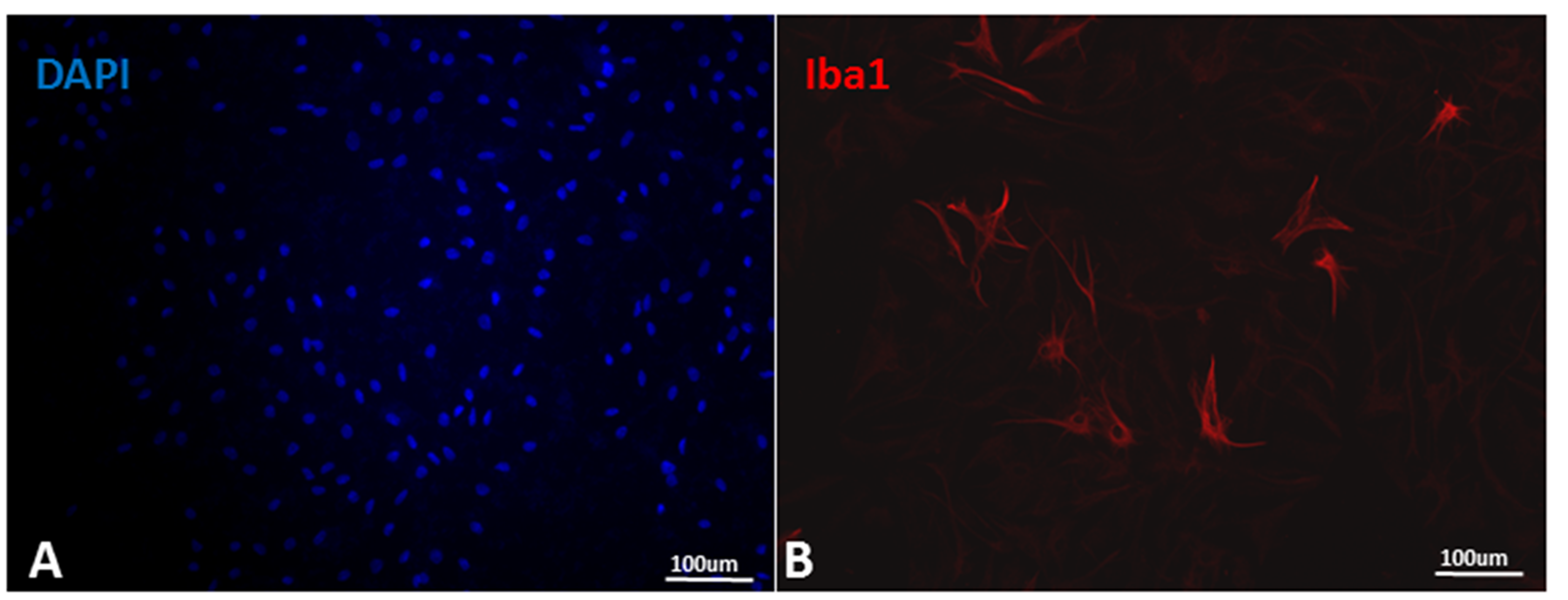

Supplement: Figure S1 — NSC monolayers contain a subpopulation of cells reactive to antibodies against the microglial marker, Iba1. The images shown are representative of an analysis of three experiments. (TIF) [file pone.0034097.s001.tif]

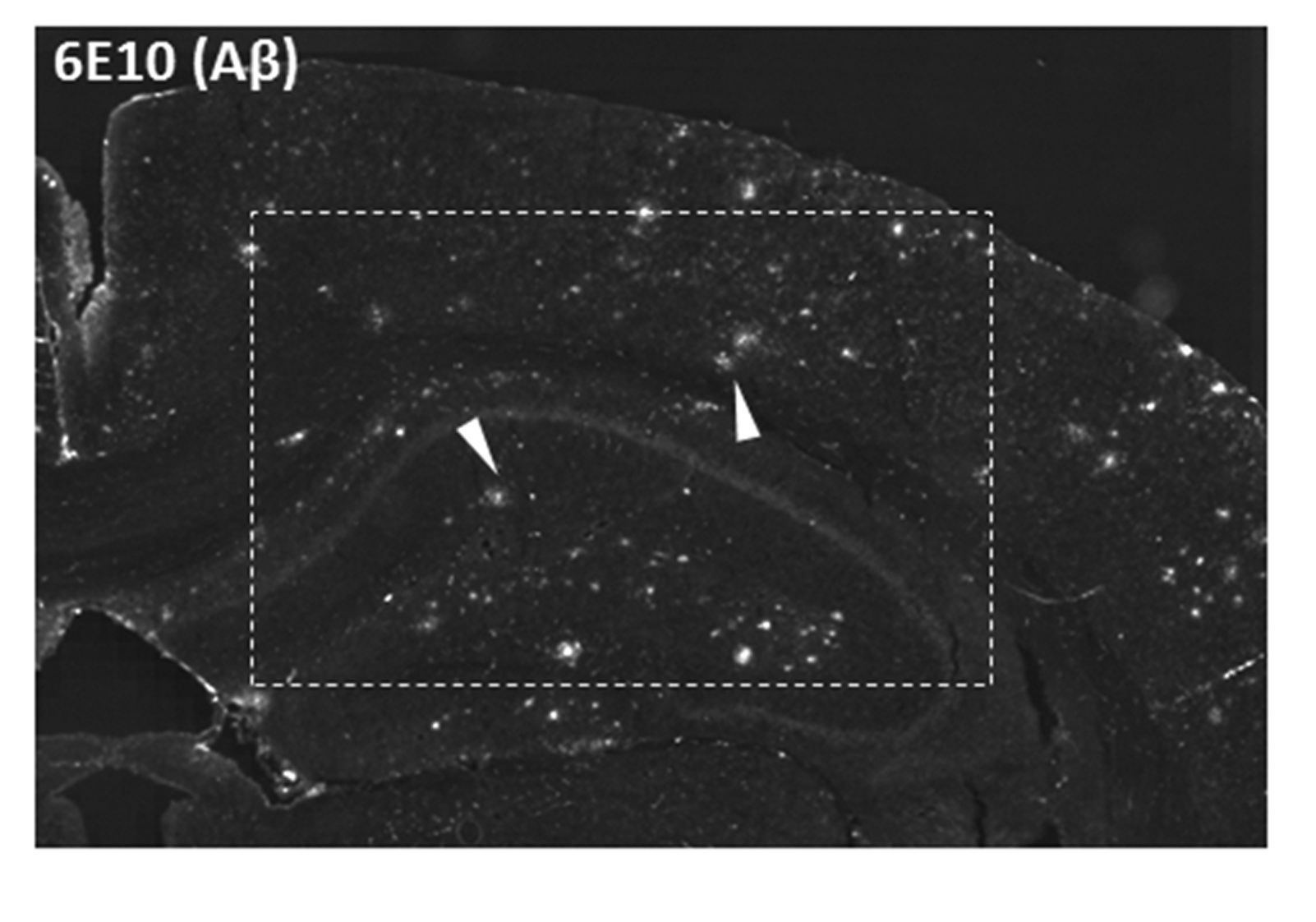

Supplement: Figure S2 — Quantification of Aβ plaque burden in APPswe/PS1dE9 mice. A defined area of consistent size (ROI: 1.25 mm×2 mm) was outlined on each hemisphere, encompassing part of the cortex and hippocampus. 6E10 immunoreactive Aβ plaques (arrowheads) within this region were counted and compared. (TIF) [file pone.0034097.s002.tif]

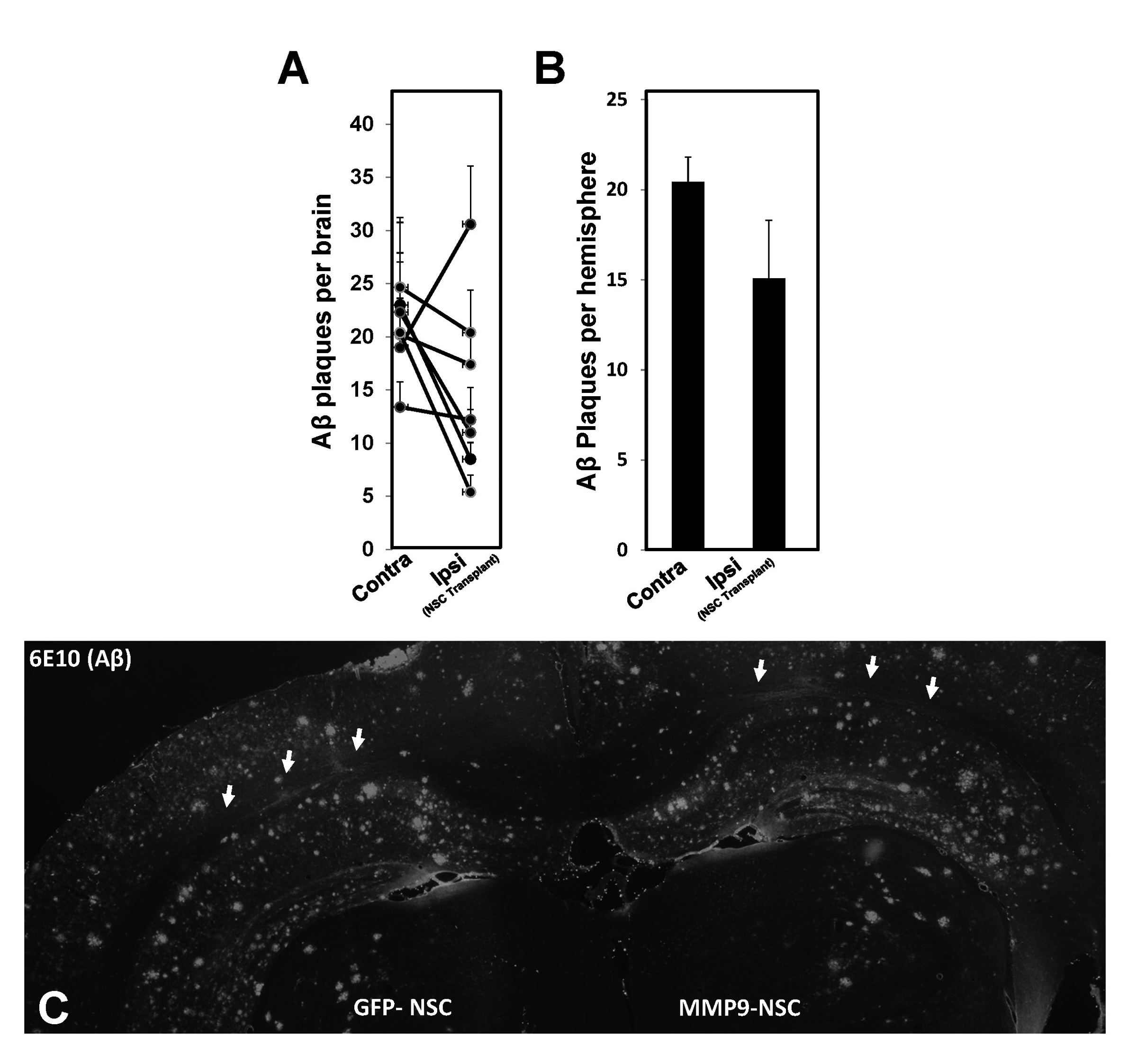

Supplement: Figure S3 — Graft associated reduction of Aβ plaques in tetAPPsi mice. TetAPPsi mice on doxycycline diet to inhibit new Aβ deposition hosted MMP9-NSCs in one hemisphere for two months. Transplantation was associated with less plaques in all but one animal (A, n = 7). Across all mice, grafts were associated with 26.1% less Aβ plaques (B). The number of Aβ plaques in a graft-associated ROI of 0.25 mm×0.35 mm were counted with NIH Image J (see Methods). In follow-up experiments, GFP-NSCs and MMP9-NSCs were bilaterally engrafted for a month. No obvious differences were observed in this side-by-side test on the effect of NSC derived MMP9 on pre-existing Aβ deposits (C, representative image from n = 9, arrows mark the position of the grafts). (TIF) [file pone.0034097.s003.tif]

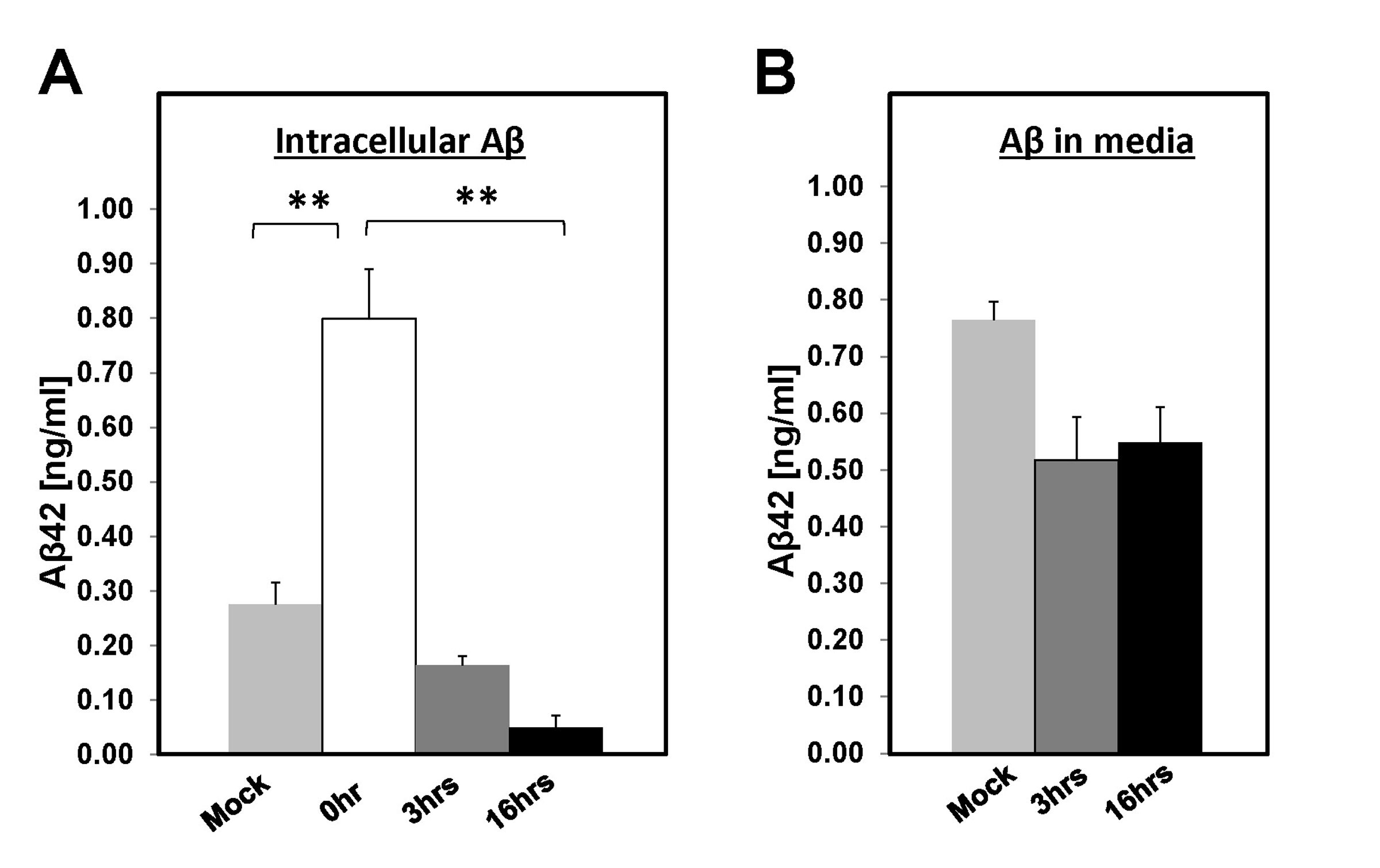

Supplement: Figure S4 — NSCs possess an inherent capacity to degrade Aβ42 peptides. Cultured NSCs were bathed in media containing 4 µg/mL of Aβ42 (n = 4) before we subsequently assayed Aβ42 levels in cell lysates and culture medium at 3 and 16 hours later by ELISA. The medium was analyzed to determine whether any peptide taken up by these cells was recycled back to medium. In all cases we included a sample in which no cells were present in the culture vessel to control for non-specific binding of Aβ to plastic. Note that there is significant nonspecific adherence of the peptide to plastic, and significant re-solubilization occurs when medium is reapplied to the vessel. Despite the unavoidable problems with non-specific binding of Aβ to culture vessels, we observed a very significant increase in Aβ levels in cell lysates after incubation with levels quickly falling back to baseline levels (A). As Aβ levels in cell lysates declined, we did not observe a parallel increase in Aβ levels in medium above what occurs from leaching from the plastic (B 3 hrs, p<0.01; 16 hrs, p<0.01). Therefore, we conclude that NSCs were degrading the internalized peptides. Statistical analyses were performed as described in Materials and Methods: *,p<0.05; **, p<0.01. (TIF) [file pone.0034097.s004.tif]

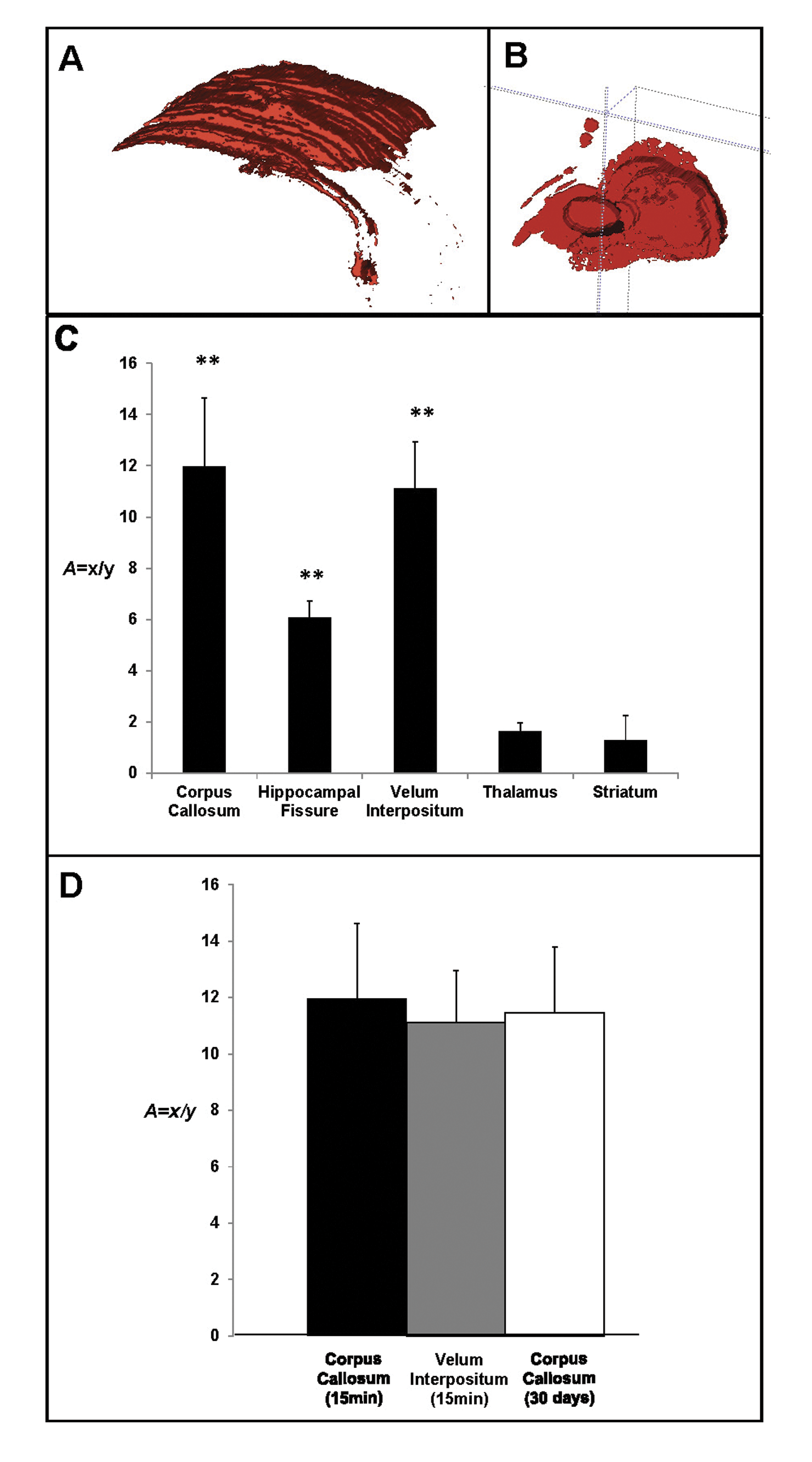

Supplement: Figure S5 — Geometric analyses of short-term NSC engraftments. 3D reconstruction of serial sections shows engraftments form a sheet-like spread in the corpus callosum (A) and a globular spread in the thalamus (B). To distinguish these differences numerically, the travel distance of NSCs in the transverse plane (x) was divided by the travel distance in the vertical plane (y), yielding an anisotropic value, A. A is 7.3× larger in the corpus callosum compared to the thalamus (C). Similar comparison indicates A values of the hippocampal fissure and velum interpositum are 3.7× and 6.8×, respectively, to the thalamus (**, p<0.01, comparison to thalamus). Comparison of the index of anisotropy of long-term engraftments in the corpus callosum to short-term engraftments in the corpus callosum and velum interpositum reveals near identical values (D), indicating month-long engraftments in AD mice are anisotropic. The anisotropic values were calculated from an analysis of a minimum of three infusions for each structure indicated. Anisotropic values for long-term engraftments were calculated from APPswe/PS1dE9 mice. (TIF) [file pone.0034097.s005.tif]

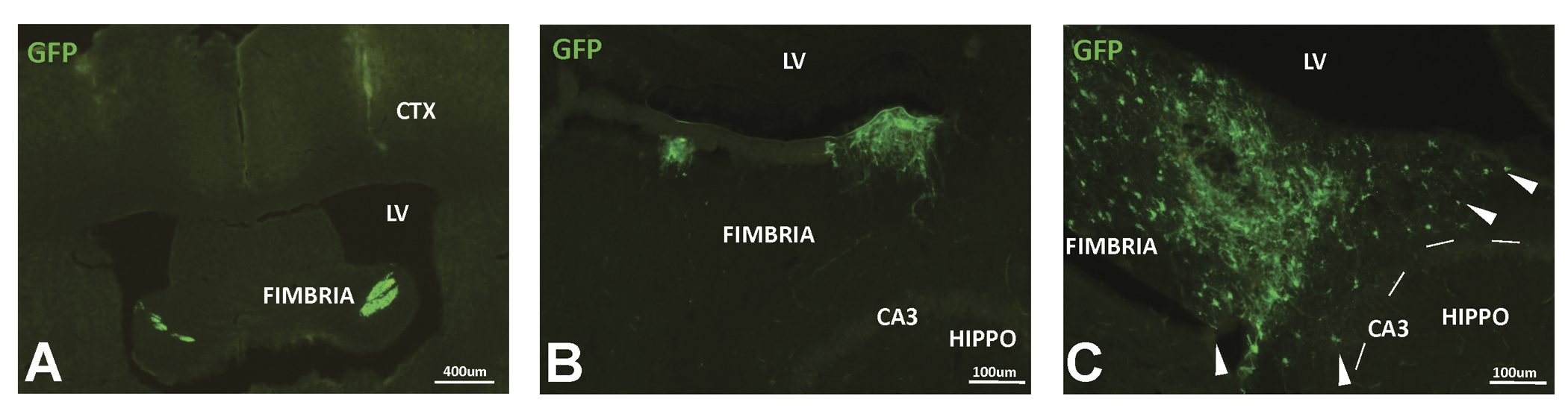

Supplement: Figure S6 — Engraftment in white matter results in permanent localization of NSCs in white matter. We infused NSCs into the fimbria, a white matter structure along the medial edge of the hippocampus. NSCs infused into the fimbria are positioned to migrate into the hippocampus, the thalamus or enter the lateral ventricle. Non-transgenic mice were used to avoid the possibility of effects specific only to AD pathology. Observation of NSCs shortly after infusion (<15 min) revealed graft cores of cells in close association of each other within the fimbria (A, n = 6). On the other hand, cells that were engrafted for one year migrated within the fimbria to the walls of the lateral ventricle and near CA3 (B–C, n = 3). No cells were found in the hippocampus or the thalamus (C, arrowheads indicate migrating cells, dashes mark hippocampal border). Thus in our models, NSCs migrated within white matter but did not exit to surrounding structures. (TIF) [file pone.0034097.s006.tif]
